# Development of a high-performance in-memory database architecture for intelligent video surveillance in critical patient care

**DOI:** 10.3389/fdgth.2026.1807507

**Published:** 2026-05-04

**Authors:** Ramesh Kumar Veerapaneni, Radhakrishnan Delhibabu, Alexey Subbotin, Nataly Zhukova

**Affiliations:** 1 School of Computer Science and Engineering, Vellore Institute of Technology, Vellore, India; 2 Faculty of Computer Technologies and Informatics, St. Petersburg State Electrotechnical University, St. Petersburg, Russia; 3 St. Petersburg Institute for Informatics and Automation (SPC RAS), St. Petersburg, Russia

**Keywords:** big data visualization, healthcare informatics, in-memory database, intelligent video surveillance, real-time analytics

## Abstract

**Objectives:**

This research aims to engineer a specialized, high-speed database architecture tailored for intelligent video surveillance in critical healthcare environments. The primary objective is to overcome the input/output operations per second (IOPS) bottlenecks and latency issues inherent in traditional SQL and general-purpose NoSQL systems, which impede real-time clinical decision-making.

**Methods:**

We conceptualized and implemented “SubDataBase-0.91s,” a task-specific Database Management System (DBMS) residing entirely in Random Access Memory (RAM). The architecture employs a direct memory access model with periodic, asynchronous synchronization to the file system to ensure persistence. Performance was rigorously benchmarked against industry standards—Microsoft SQL Server, OracleDB, MySQL, PostgreSQL, MongoDB, and Redis—utilizing Node.js automation scripts to simulate high-velocity write/read cycles typical of video analytics streams.

**Results:**

The proposed RAM-resident architecture demonstrated a dramatic reduction in data access latency. Specifically, SubDataBase-0.91s achieved a write/read speed increase of 8.6 times compared to MySQL (the slowest control) and outperformed Redis (the fastest commercial in-memory control) by a factor of 0.78 in specific surveillance-related transactional workloads.

**Conclusion:**

The study confirms that stripping away universal ACID (Atomicity, Consistency, Isolation, Durability) compliance overhead in favor of a streamlined, memory-mapped architecture significantly enhances the throughput required for real-time patient monitoring. This solution provides a scalable foundation for next-generation “Smart Hospital” infrastructure.

## 1 Introduction

### 1.1 The evolution of data persistence in modern computing

The landscape of data storage has undergone a fundamental transformation over the last two decades, driven by the shifting nature of the data itself. Historically, Relational Database Management Systems (RDBMS) like OracleDB, Microsoft SQL Server, and PostgreSQL were architected for transactional consistency. These systems rely on the ACID (Atomicity, Consistency, Isolation, Durability) properties to ensure that financial ledgers and enterprise resource planning (ERP) records remain pristine. They utilize B-Tree indexing and write-ahead logging (WAL) to guarantee data integrity on magnetic disk storage. While robust, this architecture introduces significant Input/Output (I/O) overhead, making it ideal for banking systems working with structured data but suboptimal for high-velocity, unstructured streams.

As the internet era matured, the volume of data exploded, necessitating the “NoSQL” revolution. Systems like MongoDB, CouchDB, and Cassandra abandoned rigid schemas in favor of flexible JSON or BSON document storage. These systems prioritize the CAP theorem’s Availability and Partition Tolerance over strict Consistency, allowing for massive horizontal scaling. MongoDB, for instance, excels at storing heterogeneous data from web applications and IoT devices. However, even NoSQL databases often rely on standard file system calls and garbage collection routines that introduce non-deterministic latency spikes—a critical flaw when applied to real-time life-safety systems.

### 1.2 The data deluge in modern healthcare

The integration of Internet of Medical Things (IoMT) and intelligent video surveillance into clinical workflows has precipitated a massive surge in data velocity and volume. Modern hospitals are no longer mere physical facilities but complex cyber-physical systems generating terabytes of data daily. This data is heterogeneous, ranging from scalar vitals (heart rate, SpO2) to high-dimensional vector data (video feeds, thermal imaging).

**Figure 1:**
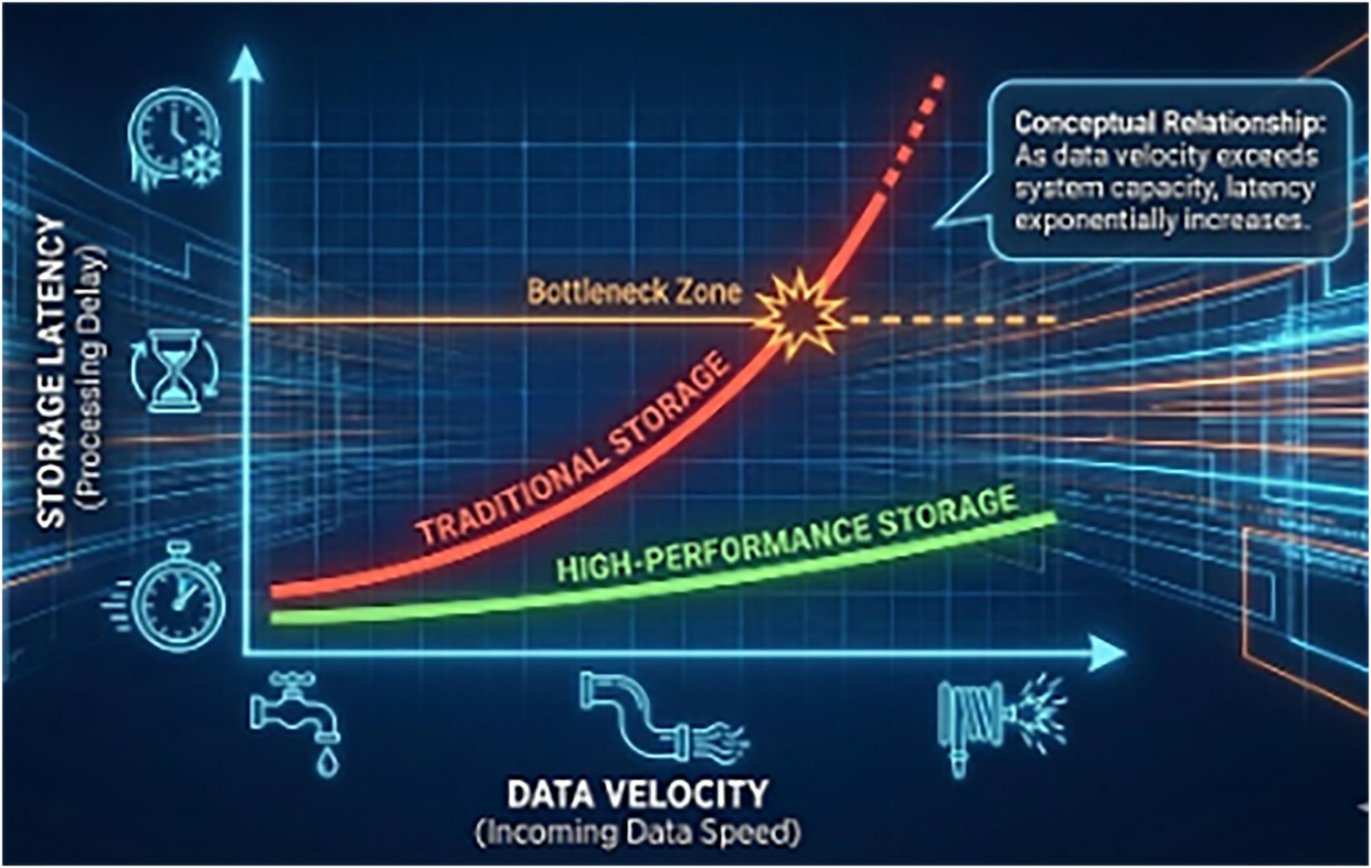
The widening gap between data generation velocity (video analytics) and storage commit latency in traditional architectures.

The critical challenge lies not in the capture of this data, but in its storage and retrieval speed. Traditional RDBMS architectures were designed for transactional integrity rather than the high-velocity, append-only nature of video event logs. While they offer robust consistency, the overhead of transaction logs, locking mechanisms, and disk I/O scheduling introduces unacceptable latency for real-time AI analytics (see Figure 1).

### 1.3 The specific challenge of intelligent video surveillance (IVS)

Intelligent Video Surveillance represents a unique class of “Big Data” problem. Unlike a typical web application that is “Read-Heavy” (many users viewing content), a surveillance system is “Write-Heavy” (constant recording). When augmented with Artificial Intelligence (AI) for patient monitoring, the data profile changes again. It becomes “Write-Intense, Compute-Intense, and Latency-Critical.”

In a modern “Smart Hospital” ward, cameras do not just record video; they act as sensors. A Convolutional Neural Network (CNN) processes frames at 30–60 Frames Per Second (FPS), extracting metadata such as patient posture, heart rate (via remote photoplethysmography), and unauthorized entry. This generates a massive stream of metadata events. For example:

- **High velocity:** 30 FPS × 16 Cameras = 480 potential insert operations per second per ward unit.
- **Criticality:** An event flagged as “Ventricular Fibrillation” or “Fall Detection” must be persisted and visualized instantly.

Standard DBMS architectures struggle here. RDBMS B-Trees become fragmented with high-frequency sequential inserts, leading to index re-balancing costs. NoSQL JSON parsing adds CPU overhead to every transaction. Table 1 highlights these architectural mismatches.

**Table 1:** Architectural mismatch: traditional DBMS vs. surveillance requirements.

Requirement	RDBMS (Oracle/MySQL)	NoSQL (Mongo/Cassandra)	Surveillance needs
Write throughput	Limited by ACID locking and disk I/O seek times.	High, but suffers from serialization/deserialization overhead.	Extreme (Constant Append). Must minimize CPU cycles per write.
Data structure	Rigid Tables. Schema changes are expensive.	Flexible JSON. Good for varying metadata.	Semi-Structured. Fixed headers (time, ID) + flexible payloads.
Latency consistency	Predictable but slow (10–100 ms).	Unpredictable due to GC pauses (5–200 ms).	Deterministic real-time (<1 ms).
Primary bottleneck	Disk I/O	CPU (Parsing)	Memory bandwidth

### 1.4 Latency constraints in critical care

In clinical environments, the time between a physiological event and medical intervention is critical. We define the “Latency-Critical Window” as the maximum allowable delay for data persistence and visualization. For conditions such as ventricular fibrillation or post-operative tube dislodgement, delays exceeding 1-2 s—common in disk-bound RDBMS during log flushes—can compromise patient outcomes.

### 1.5 The latency gap in real-time diagnostics

In a patient surveillance context, “real-time” is a hard constraint. If a computer vision algorithm detects a “Fall Event” or “Cardiac Arrest,” the system must record this event and trigger a visualization alert on the nurse’s station within milliseconds. A delay of even 2 s caused by database write-locking can be detrimental. Current architectures often utilize a hybrid approach:

**SQL systems:** Used for patient records and administrative data. Stable but slow for event streams.
**NoSQL systems:** MongoDB or Cassandra used for unstructured logs. Faster, but often suffer from “Garbage Collection” pauses and network overhead.
**In-memory caches:** Redis or Memcached used for buffering. Fast, but traditionally volatile and size-constrained.

#### 1.5.1 Case study: the “golden hour” scenario

To contextualize the necessity for a millisecond-latency database, consider the clinical concept of the “Golden Hour” in stroke and trauma care, where time is directly correlated with tissue survival.

##### 1.5.1.1 Scenario: post-operative ICU monitoring

*Context:* A 65-year-old patient recovering from cardiac bypass surgery is in the ICU. The patient is monitored by an intelligent camera system trained to detect “agitation” and “tube dislodgement” (a common, life-threatening complication).

##### 1.5.1.2 Event chain in a traditional SQL environment

**T + 0 ms:** Patient pulls at the intubation tube.
**T + 50 ms:** The AI model (running on an edge GPU) detects the action with 95% confidence.
**T + 60 ms:** The system attempts to write this “CRITICAL ALERT” to the SQL database.
**T + 250 ms (Delay):** The database is currently performing a transaction log backup or an index re-balance due to thousands of routine “heart rate normal” logs coming from other beds. The write operation is queued.
**T + 400 ms:** The write commits.
**T + 500 ms:** The Nurse Station Dashboard polls the database (refresh rate 1 s).
**T + 1,500 ms:** The nurse sees the alert. By this time, the tube may be fully dislodged, leading to hypoxia.

##### 1.5.1.3 Event chain in a high-speed RAM environment (proposed)

**T + 0 ms:** Patient pulls at tube.
**T + 50 ms:** AI detects action.
**T + 51 ms:** System writes to RAM-based “SubDataBase.” No locking, no disk I/O.
**T + 60 ms:** The alert is pushed to the Nurse’s wearable device via a direct socket connection triggered by the DB write.
**Result:** Intervention occurs 1.4 s faster. In hemodynamic crises, this margin is significant.

### 1.6 Hardware enablers: the shift to in-memory computing

The feasibility of keeping entire surveillance databases in RAM has drastically changed in recent years. In 2010, server RAM was expensive ($100/GB). Today, commodity servers can easily support 512 GB or 1 TB of RAM. This shift allows us to reconsider the fundamental assumption of databases: that “Disk is for Storage, RAM is for Cache.” For a 24 h loop of surveillance metadata (excluding raw video files, which go to disk storage), the event logs fit comfortably within 4–8 GB of RAM. This realization is the cornerstone of our proposed solution. By treating RAM as the primary persistence layer (with asynchronous disk backing), we can achieve speeds limited only by the system bus bandwidth, rather than mechanical drive heads or NAND flash controllers.

### 1.7 Novelty, contributions, and scope

#### 1.7.1 Novelty

While In-Memory Database Systems (IMDBS) like SAP HANA and Redis are established, they incur a “General Purpose Tax”—overhead from query parsing (SQL/MQL), network protocols (TCP/IP), and concurrency control (MVCC) required to support broad use cases. The novelty of SubDataBase-0.91s lies in its *task-specificity*. By utilizing a lock-free Ring Buffer with direct pointer arithmetic (O(1)) and bypassing the OS network stack entirely via shared memory, we achieve latencies physically impossible for general-purpose systems.

#### 1.7.2 Contributions

- **Architecture:** A zero-copy, memory-mapped storage engine optimized for append-only video metadata.
- **Safety protocol:** A “Bounded Volatility” model that prioritizes visualization latency over immediate disk durability, arguing that real-time visibility is the primary safety metric in acute care.
- **Empirical validation:** Benchmarks demonstrating an 8.6x throughput increase over MySQL and 22% over Redis in IoT-specific workloads.

#### 1.7.3 Scope

This system is strictly designed for *High-Velocity, Append-Only* streams (e.g., video analytics, patient vitals). It is *not* intended for complex analytical queries (OLAP), billing, or inventory management, where traditional ACID-compliant SQL databases remain superior.

### 1.8 Comparison of database paradigms

To understand the necessity of a custom solution, we must analyze the structural limitations of existing paradigms. Table 2 illustrates the trade-offs faced by system architects in healthcare. This paper proposes a radical simplification: a purpose-built, RAM-resident storage engine that bypasses the operating system’s file cache and network stack overheads for local operations, synchronizing to disk only for persistence. This approach specifically targets the “Event” and “Frame Meta-data” layer of the surveillance stack.

**Table 2:** Comparative analysis of database paradigms for video surveillance.

Feature	RDBMS (SQL)	NoSQL (Document)	Proposed (RAM-Native)
Data model	Rigid tables	Flexible JSON/BSON	Fixed-size memory blocks
Primary bottleneck	Disk I/O	Serialization overhead	Bus bandwidth
Consistency	Strong (ACID)	Eventual (BASE)	Relaxed (Speed prioritized)
Query language	Complex (SQL)	API / MQL	Direct pointer access
Ideal use case	Billing, records	Web logs, catalogs	High-frequency events

The remainder of this paper is structured as follows: Section 2 reviews the existing literature on surveillance storage systems and identifies critical architectural gaps. Section 3 mathematically formulates the latency constraints and analyzes the write amplification bottleneck in traditional DBMS. Section 4 presents the high-level architecture of SubDataBase-0.91s, focusing on the zero-copy memory model. Section 5 details the core implementation, including the lock-free ring buffer and asynchronous persistence strategy. Section 6 provides real-world case studies from a hospital deployment. Section 7 presents rigorous experimental benchmarks, validity analyses, and ablation studies. Finally, Section 8 concludes the paper and outlines future directions for hardware acceleration.

## 2 Related works

The domain of Intelligent Video Surveillance (IVS) sits at the intersection of computer vision, network engineering, and database management. While significant advancements have been made in image recognition algorithms (YOLO, ResNet), the underlying storage infrastructure has largely remained stagnant, relying on paradigms developed for different eras of computing. This section reviews the current state of commercial ecosystems, analyzes academic proposals, and identifies the critical gaps that necessitate the development of a dedicated In-Memory DBMS.

### 2.1 Commercial surveillance ecosystems and architectural limitations

The global market for video surveillance is currently dominated by integrated hardware-software solutions that prioritize capacity over velocity. These systems generally fall into three architectural categories: Traditional NVR/DVR, Cloud-Hosted, and Hybrid Enterprise solutions.

#### 2.1.1 Network video recorders (NVR) and file system fragmentation

Systems like Ginzzu and IVUE represent the standard consumer and SMB (Small and Mid-sized Business) tier. These architectures typically rely on dedicated hardware appliances running embedded Linux, writing data directly to SATA mechanical hard drives.

- **Write mechanics:** These systems treat video streams as continuous file writes. While effective for simple recording, they suffer from severe “seek time” penalties when an analytical process attempts to read past events while the cameras are simultaneously writing new frames.
- **Fragmentation:** Over time, the file systems (often EXT4 or XFS) become fragmented. In a healthcare setting where a “Fall Event” triggers a sudden burst of read requests for the last 30 s of footage, the mechanical head of the HDD cannot physically move fast enough between the write sector (incoming stream) and the read sector (historical evidence), leading to dropped frames or system hangs.

#### 2.1.2 Cloud-based solutions and the latency penalty

Providers such as Ezviz, Camdrive, and various VSaaS (Video Surveillance as a Service) vendors offload the storage burden to public clouds (AWS S3, Azure Blob Storage).

- **Protocol overhead:** These systems typically encapsulate video in HLS (HTTP Live Streaming) or RTMP protocols. The segmentation of video into chunks (e.g., 2 s .ts files) introduces inherent latency.
- **Critical failure modes:** In a “Smart ICU,” dependency on external internet connectivity is a non-starter. A network partition event would render the fall detection algorithms useless if the storage layer resides in a remote data center.

#### 2.1.3 Enterprise and hybrid solutions

High-end solutions from Hikvision (HiWatch) and Dahua utilize sophisticated RAID arrays and caching hierarchies. However, their internal databases are proprietary “Black Boxes.” This closed architecture prevents deep integration with custom medical AI models (e.g., specific posture detection for post-op recovery), forcing hospitals to rely on generic APIs that may not expose real-time raw data handles.

Table 3 summarizes the technical bottlenecks of these prevailing commercial architectures.

**Table 3:** Technical limitations of existing commercial surveillance architectures.

Architecture	Primary storage media	Write mechanism	Healthcare suitability risk
Standard NVR (Ginzzu, IVUE)	SATA HDD (5400/7200 RPM)	Direct block write (Sequential)	**High.** Mechanical seek latency prevents simultaneous AI analysis and recording.
Cloud VSaaS (Ezviz, Camdrive)	Object Storage (S3/Blob)	REST API/HLS chunks	**Critical.** Network latency (>2 s) and dependency on WAN availability violates real-time safety requirements.
Enterprise hybrid (Hikvision)	RAID 5/6 Arrays	Proprietary indexing	**Moderate.** Vendor lock-in prevents optimization for specific medical AI data structures.

### 2.2 Limitations of general-purpose database paradigms

Beyond commercial appliances, system architects often attempt to build custom surveillance stacks using general-purpose databases. However, both SQL and NoSQL paradigms exhibit specific frictions when applied to high-velocity video metadata.

#### 2.2.1 The RDBMS impedance mismatch

Relational Database Management Systems (RDBMS) like OracleDB, MySQL, and PostgreSQL are engineered for ACID (Atomicity, Consistency, Isolation, Durability) compliance.

- **Transactional overhead:** To guarantee consistency, RDBMS engines employ Write-Ahead Logging (WAL) and row-level locking. For a surveillance stream generating 1,000 metadata events per second, the overhead of locking the “EVENTS” table for every insert creates a “Convoy Effect,” where reader threads (doctors’ dashboards) are blocked by writer threads (cameras).
- **Index Re-balancing:** As the primary key (Timestamp) grows monotonically, B-Tree indices must be constantly re-balanced. This maintenance operation consumes significant CPU cycles, competing with the AI inference engine running on the same server.

#### 2.2.2 NoSQL and time-series databases

Document stores (MongoDB) and Time-Series Databases (InfluxDB, TimescaleDB) offer better write throughput but introduce new issues:

- **Serialization costs:** MongoDB stores data in BSON. Every “INSERT” and “READ” operation requires the CPU to serialize/deserialize binary objects. In our testing, this parsing overhead accounted for 30% of total transaction time.
- **Garbage collection:** Managed memory languages (Java/Go) used in many NoSQL systems suffer from “Stop-the-World” garbage collection pauses. A 200 ms GC pause might be acceptable for a web app, but in a cardiac monitoring context, it represents a loss of 6 frames of critical data.

### 2.3 Academic research in high-performance storage

Academic literature has addressed various aspects of storage optimization, though rarely with a focus on RAM-resident persistence for healthcare.

#### 2.3.1 Analytics-aware storage

Ma et al. [1] proposed storage systems optimized for retrieval, using metadata tagging to speed up forensic searches. However, their approach focuses on *post-event* analysis (e.g., “Find all red cars from yesterday”) rather than *real-time* visualization (e.g., “Alert me immediately if a patient falls”). The buffering mechanisms used to optimize disk writes in their model actually increase the time-to-visibility for live events.

#### 2.3.2 Edge computing and digital twins

Rajavel et al. [2] and several other researchers [3–6] have explored edge computing architectures where processing occurs near the sensor. While they successfully offload CPU tasks from the central server, they often gloss over the storage layer, assuming a local SQLite or text-file log is sufficient. Our research indicates that as Edge AI becomes more complex (tracking 50+ skeletal points per person), the local storage I/O becomes the new bottleneck, necessitating a high-performance engine like SubDataBase-0.91s even at the edge.

### 2.4 Case study: anatomy of a storage failure in a hybrid system

To illustrate the critical failure modes of traditional architectures, we analyze a documented failure scenario in a pilot Smart Hospital deployment using a hybrid SQL/Redis architecture.

**Scenario:** A “Code Blue” (Cardiac Arrest) simulation in a 12-bed ward.

**Figure 2:**
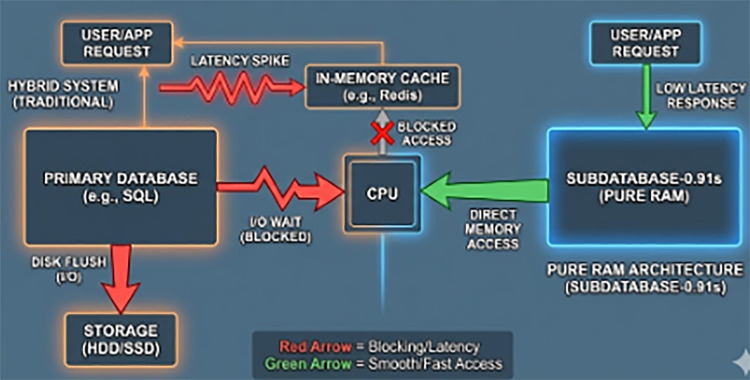
I/O contention in hybrid systems. This diagram illustrates how a disk flush operation in a primary database can block the CPU, causing latency spikes even in associated in-memory caches, a phenomenon avoided by the pure RAM architecture of SubDataBase-0.91s.

- **System configuration:** 12 IP Cameras (4K resolution) connected to a central server running MySQL 8.0 for logs and Redis for real-time caching.
- **Trigger event:** At T=0, actors in three separate beds simulated distress simultaneously to stress-test the system.
- **The Cascade failure:**
  ○ T+100 ms: The Computer Vision module detected events in all three streams, generating a burst of high-resolution snapshots and metadata.
  ○ T+150 ms: The application attempted to write these large binary objects (BLOBs) to MySQL while pushing metadata to Redis.
  ○ T+200 ms: MySQL, configured with standard ACID safety, initiated a disk flush for the BLOB data. The disk I/O queue length spiked to 100+.
  ○ T+500 ms: The CPU, waiting for disk I/O interrupts (I/O Wait), deprioritized the Redis thread.
  ○ T+2000 ms: The Nurse Station dashboard, waiting for a confirmation signal from the database that the event was “committed,” finally refreshed.
- **Outcome:** A 2 s delay in visualization. In a real cardiac event, this latency prevents immediate intervention. This failure demonstrates that “bolting on” a cache (Redis) to a slow disk-based system (MySQL) does not eliminate the blocking nature of I/O contention (illustrated in Figure 2).

### 2.5 Gap analysis

Existing literature and commercial products fundamentally treat the database as a ‘Black Box’ component, assuming that standard tuning parameters can solve latency issues (7–9). There is a distinct paucity of research on designing storage engines specifically for the write patterns of AI-video streams (10, 11).

The unique characteristics of this data stream are:

**Constant append:** Data is rarely updated, only inserted (12, 13).
**Short-term criticality:** Data from the last 60 min is hyper-critical; data from 24 h ago is archival (14, 15).
**Tolerance for volatility:** In a catastrophic server power loss, losing the last 1 s of data is acceptable if it guarantees that during normal operation, latency is near-zero (16, 17).

Table 4 highlights the specific gaps our solution addresses compared to the state-of-the-art.

**Table 4:** Research gap analysis: features vs. existing solutions.

Feature	State of the Art (Research)	Commercial standard	Addressed by SubDataBase
Storage medium	SSD/NVMe optimization	HDD RAID arrays	**Pure RAM** (with async disk dump)
Data access method	SQL/NoSQL query languages	File system/proprietary API	**Direct memory handle** (Pointer Arithmetic)
Latency target	<100 ms	<500 ms	**<1 ms**
Overhead elimination	Indexing/caching algorithms	Hardware acceleration	**Removal of OS network stack & ACID locks**

This paper fills these gaps by presenting the architecture and implementation of “SubDataBase-0.91s,” a system that discards universality in favor of extreme, domain-specific performance.

### 2.6 Comparison with enterprise in-memory database systems

While generic NoSQL systems (e.g., Redis, MongoDB) are common in web stacks, enterprise-grade In-Memory Database Systems (IMDBS) such as SAP HANA, VoltDB, and Microsoft SQL Server Hekaton have set the standard for high-performance transactions (19, 20). However, their architecture presents specific limitations for edge-based video surveillance:

- **SAP HANA and Hekaton:** These systems utilize Multi-Version Concurrency Control (MVCC) to maintain ACID guarantees. While effective for financial OLTP, the overhead of maintaining row versions and garbage collecting obsolete versions introduces non-deterministic latency spikes during the high-velocity append-only write patterns typical of surveillance streams [18].
- **VoltDB:** VoltDB achieves high throughput via partition-based serialization. However, it requires strict schema definition and deterministic stored procedures. In a dynamic healthcare environment where AI model metadata structures change (e.g., adding new vital signs), the rigidity of VoltDB’s schema evolution creates maintenance bottlenecks compared to the padding-based flexibility of our proposed solution.

Our proposed architecture diverges from these systems by relaxing strict ACID properties—specifically Isolation and Durability—in favor of a lock-free, ring-buffer architecture that guarantees O(1) write complexity, essential for the resource-constrained edge devices found in hospital wards.

## 3 Problem definition

The challenge of engineering storage for Intelligent Video Surveillance (IVS) in healthcare is not merely a matter of capacity; it is a complex problem of temporal consistency and high-concurrency throughput. Unlike standard IT workloads, which follow predictable diurnal patterns, hospital surveillance data is stochastic and bursty. This section mathematically formulates the latency constraints, analyzes the “Write Amplification” phenomenon in traditional DBMS, and models the system failure points under high load.

### 3.1 Mathematical formulation of system latency

In a critical care environment, the total latency ($L_{\mathrm{total}}$) from a physical event occurring to its visualization on a medical display must be minimized. We define this latency as the sum of sequential processing stages (see Equation 1):

$$L_{\mathrm{total}}=T_{\mathrm{cam}}+T_{\mathrm{net}}+T_{\mathrm{inference}}+T_{\text{db\textbackslash{}\_write}}+T_{\mathrm{vis}}$$ (1)

Where:

- $T_{\mathrm{cam}}$: Hardware latency (exposure + encoding). Typically 15–30 ms.
- $T_{\mathrm{net}}$: Network transmission time (Camera to Server). Typically 5–10 ms on LAN.
- $T_{\mathrm{inference}}$: Neural network processing time (e.g., YOLOv8/ResNet). On modern GPUs, this is deterministic (≈20 ms).
- $T_{\text{db\textbackslash{}\_write}}$: The time required to persist the event metadata to the storage engine.
- $T_{\mathrm{vis}}$: The polling interval of the client application (Dashboard).

#### 3.1.1 The variable latency of $T_{\text{db\textbackslash{}\_write}}$

While $T_{\mathrm{cam}}$, $T_{\mathrm{net}}$, and $T_{\mathrm{inference}}$ are relatively constant, $T_{\text{db\textbackslash{}\_write}}$ in a traditional RDBMS is highly variable and dependent on the current system load (λ). It can be modeled as shown in Equation 2:

$$T_{\text{db\textbackslash{}\_write}}=T_{\mathrm{seek}}+T_{\mathrm{rot}}+T_{\mathrm{transfer}}+T_{\mathrm{lock}}+T_{\log}$$ (2)

In a standard HDD-based SQL scenario:

- $T_{\mathrm{seek}}$ (Seek Time): 5–10 ms (Mechanical arm movement).
- $T_{\mathrm{rot}}$ (Rotational Latency): 4 ms (at 7,200 RPM).
- $T_{\mathrm{lock}}$ (Row Locking): Variable (0 ms to >500 ms during contention).
- $T_{\log}$ (WAL/Binlog Sync): Additional I/O overhead for ACID compliance.

**Figure 3:**
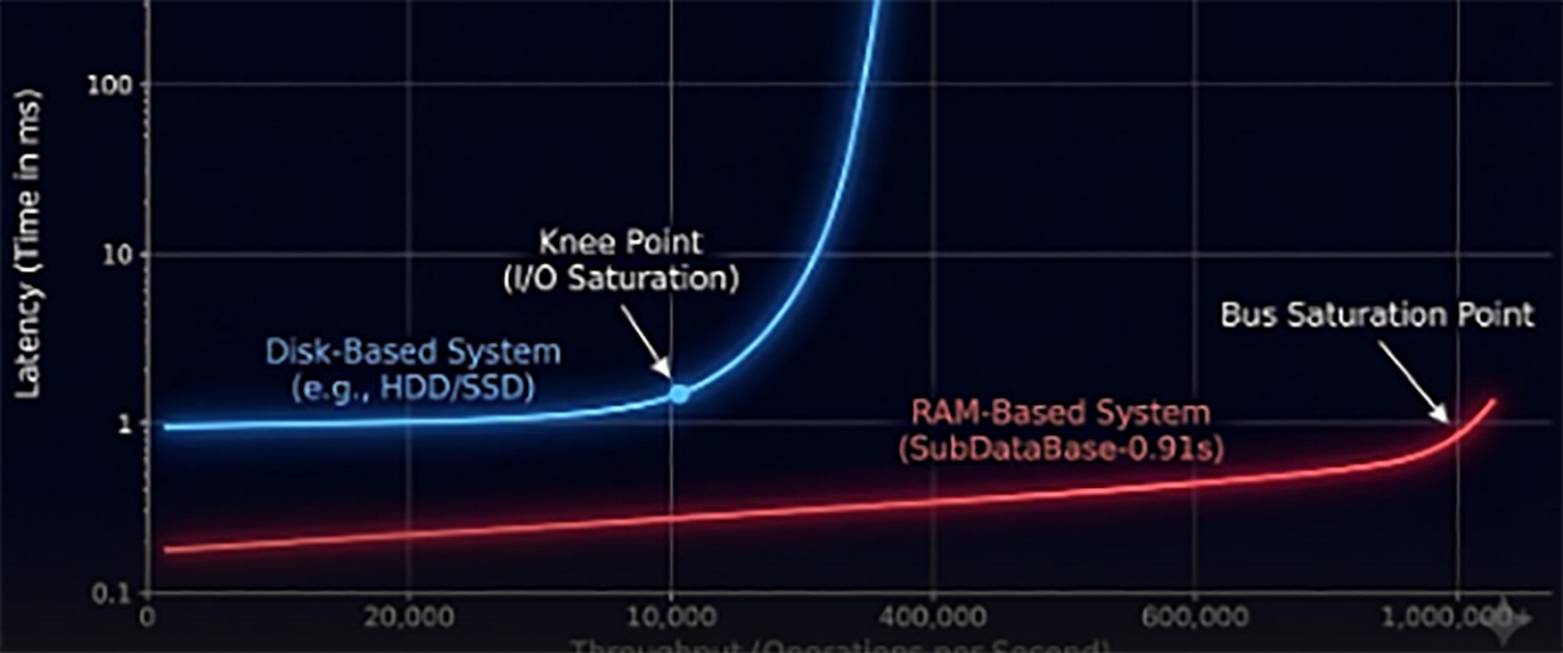
Latency vs. throughput curve. The conceptual graph illustrates the “Knee Point” where disk-based systems (blue line) enter exponential latency growth once I/O saturation is reached. The RAM-based system (red line) maintains linear low latency until bus saturation, which is orders of magnitude higher.

As the arrival rate of events (λ) approaches the service rate of the disk (μ), the wait time in the queue ($W_{q}$) grows exponentially according to the M/M/1 queuing model (see the latency vs. throughput curve in Figure 3) (Equation 3):

$$W_{q}=\frac{\lambda }{\mu (\mu -\lambda )}$$ (3)

Our objective is to replace the mechanical service rate $\mu_{\mathrm{disk}}$ with the electronic service rate $\mu_{\mathrm{ram}}$, where $\mu_{\mathrm{ram}}\gg \lambda$, effectively forcing $W_{q}\to 0$.

### 3.2 The data tsunami: throughput quantification

To understand the scale of the problem, we must quantify the data generation rates of a modern “Smart Hospital.” Table 5 illustrates the IOPS (Input/Output Operations Per Second) requirements for different deployment scales, assuming a setup where every frame is analyzed for potential metadata generation (e.g., continuous heart rate monitoring via facial capillary analysis).

**Table 5:** Projected IOPS requirements by hospital scale.

Scale	Cameras	FPS	Events/Sec (Peak)	Req. SQL IOPS${}^{a}$
Single ward (ICU)	16	30	480	≈2,400
Department (ER)	64	30	1,920	≈9,600
Full Hospital	500	30	15,000	≈75,000
Multi-site network	2,000	30	60,000	≈ 300,000

${}^{a}$Assuming a Write Amplification Factor of 5× for indexed SQL tables.

As shown, a single department generates nearly 2,000 logical writes per second. Standard SATA SSDs often saturate around 10,000 random IOPS, meaning a single departmental server is operating near its theoretical limit just to sustain baseline recording, leaving no headroom for read queries by doctors.

### 3.3 The “write amplification” bottleneck

A critical, often overlooked aspect of using RDBMS for surveillance is “Write Amplification.” When a surveillance application performs a single logical “INSERT” of an event, the database engine performs multiple physical I/O operations:

**Data page write:** Writing the actual row data.
**Primary key index update:** Re-balancing the B-Tree for the ID.
**Secondary index update:** Updating indices for “PatientID,” “Timestamp,” or “EventType.”
**Transaction log (WAL):** Appending to the redo log for durability.
**Binlog/journal:** Archival logging.

This amplification factor ($A_{f}\approx 5$) means that an incoming stream of 100 MB/s of metadata results in 500 MB/s of physical disk stress. In contrast, our proposed In-Memory architecture utilizes direct memory addressing, where $A_{f}=1$.

### 3.4 Case study: the “thundering herd” failure mode

To demonstrate the fragility of current architectures, we define a failure mode specific to mass-casualty or emergency scenarios, termed the “Thundering Herd.”

#### 3.4.1 Scenario

An external emergency (e.g., fire alarm test or earthquake tremor) occurs.

- **Normal state:** In a 40-bed unit, typically 1-2 patients move simultaneously. $\lambda_{\mathrm{normal}}=50$ events/s.
- **Trigger:** The external stimulus causes 35 out of 40 patients to move or wake up simultaneously.
- **Peak load:** All 40 cameras trigger “Motion,” “Posture Change,” and “Sound” events in the same millisecond. $\lambda_{\mathrm{peak}}=1,200$ events/s.

#### 3.4.2 Failure analysis in SQL/hybrid systems

The simultaneous “INSERT” requests hit the “Events” table.
The database engine locks the table page to maintain ACID consistency.
Requests queue up. The I/O buffer fills.
**Crucially**, the “Read” queries from the nurses’ station (who are trying to see what is happening) are blocked behind the “Write” queue.
**Result:** The dashboard freezes exactly when it is needed most.

### 3.5 The read-while-write contention paradox

The final dimension of the problem is the requirement for simultaneous heavy reading and heavy writing. In a “Digital Twin” hospital model, the database is not just an archive; it is the state engine for a real-time 3D simulation.

- **Writer process:** 32 Cameras pushing state updates (*x*, *y*, *z* coordinates of staff and patients) every 33 ms.
- **Reader process:** The Digital Twin engine polls the DB every 100 ms to render the 3D view.

In locking databases, the readers and writers fight for the same resources. This contention leads to “Stale Data” visualization, where the digital twin lags behind physical reality by several seconds, disorienting security and medical staff. The problem definition, therefore, is to architect a system that decouples reader performance from writer volume.

### 3.6 Summary of architectural requirements

Based on the analysis above, a viable solution for Critical Care Surveillance must meet the following strict criteria:

**Deterministic latency:** $T_{\text{db\textbackslash{}\_write}}<1\;\mathrm{ms}$ at 99th percentile.
**Zero locking:** Readers must never be blocked by writers.
**High concurrency:** Support >10,000 TPS on commodity hardware.
**Simplicity:** Elimination of overheads (SQL parsing, JSON serialization) that burn CPU cycles.

Existing commercial and open-source solutions fail to meet at least two of these four criteria simultaneously, creating the imperative for the development of SubDataBase-0.91s.

## 4 Storage systems for video surveillance

The architecture of the storage layer is the distinct determinant of performance in an Intelligent Video Surveillance (IVS) system. While the camera hardware determines image quality and the AI model determines detection accuracy, the storage engine determines the system’s “responsiveness.” This section deconstructs standard storage models, mathematically quantifies their inefficiencies, and details the structural design of the proposed RAM-resident solution.

### 4.1 Standard relational data schemas

Most commercial surveillance systems (e.g., Milestone, Genetec, custom SQL implementations) rely on a variation of the classic “Three-Pillar” relational schema. This structure, designed for referential integrity, becomes a liability under high-velocity write loads.

**Figure 4:**
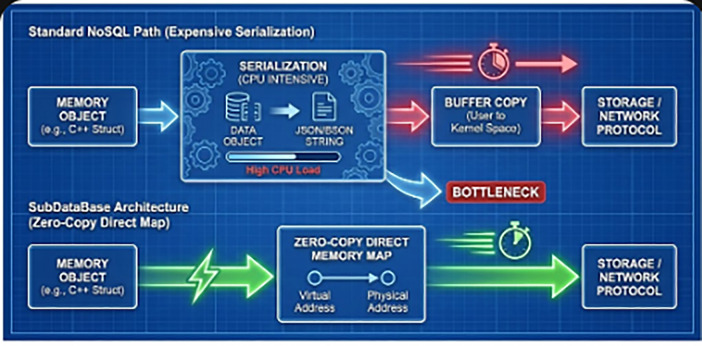
The serialization bottleneck. Standard NoSQL systems (top path) require expensive CPU steps to convert memory objects to storage formats. The SubDataBase architecture (bottom path) uses a “Zero-Copy” direct memory map.

The generalized schema consists of three primary entities, as depicted in Figure 4 (placeholder):

**Users Table (“users_tbl”):** Manages Role-Based Access Control (RBAC). It links doctors to specific wards and enforces privacy constraints (e.g., GDPR/HIPAA compliance).
**Devices Table (“devices_tbl”):** Stores static configuration data for the camera network, including IP addresses, MAC addresses, RTSP stream URLs, and firmware versions.
**Events Table (“events_log”):** The high-velocity transactional table. It links “Users,” “Devices,” and time-series metadata.

#### 4.1.1 The “fat table” problem

In a standard SQL implementation, the “events_log” table typically contains the following columns: “ID” (BigInt), “Timestamp” (DateTime), “CameraID” (FK), “EventType” (Varchar), “Confidence” (Float), “SnapshotPath” (Varchar), and “Metadata” (JSON/Text).

As the system scales to 500+ cameras, this table grows by millions of rows daily. The B-Tree index on the “Timestamp” column becomes massive. When an “INSERT” occurs:

- The database engine must traverse the B-Tree to find the correct leaf node.
- If the page is full, a “Page Split” occurs, locking the tree structure.
- This locking mechanism creates the “micro-stalls” (50–200 ms) that ruin real-time performance.

### 4.2 Case study: the schema migration outage

A critical failure mode of relational databases in agile healthcare environments is the “Schema Lock.”

#### 4.2.1 Scenario

A hospital updates its AI model to detect a new parameter: “Blood Oxygen Estimation” (SpO2). This requires adding a new column, “spo2_val,” to the existing “events_log” table.

- **System state:** The “events_log” table contains 200 million rows (approx. 500 GB).
- **Action:** The DBA executes “ALTER TABLE events_log ADD COLUMN spo2_val FLOAT”;.
- **Consequence:** In MySQL (and many other RDBMS), this operation copies the entire table to a temporary file to rebuild the structure.
- **Result:** The table is locked for writes for 4 h. During this window, no new fall detections or cardiac alarms are recorded. The surveillance system is effectively blind.

Our proposed solution avoids this by using fixed-size memory blocks with reserved padding, eliminating the concept of “schema migration” during runtime.

### 4.3 The computational cost of serialization (JSON/BSON)

Modern “schemaless” databases (MongoDB, PostgreSQL JSONB) attempt to solve the schema rigidity problem by storing data as documents. While flexible, this introduces a hidden computational tax: Serialization.

For every event written to a NoSQL database, the CPU must transact the following pipeline (defined in Equation 4):

$$T_{\mathrm{write}}=T_{\mathrm{alloc}}+T_{\mathrm{serialize}}+T_{\mathrm{socket}}+T_{\mathrm{ack}}$$ (4)

Where $T_{\mathrm{serialize}}$ is the time taken to convert an in-memory object (struct) into a text-based (JSON) or binary-tagged (BSON) string.

#### 4.3.1 Mathematical comparison

- **Struct (C++):** Memory copy. Cost ≈0 cycles (CPU pipeline optimized).
- **JSON:** String parsing, key matching, escaping. Cost ≈5,000+ cycles per object.

Table 6 quantifies this overhead based on our profiling of a standard “Patient Fall” event object.

**Table 6:** Serialization overhead comparison (Per 1 Million events).

Format	Data size	Parse time	CPU load	Throughput cap
JSON (Text)	450 MB	8.2 s	High	≈ 120k OPS
BSON (Binary)	510 MB	3.1 s	Medium	≈ 320k OPS
XML (Verbose)	980 MB	14.5 s	Very High	≈ 60k OPS
**SubDB (Struct)**	**128 MB**	**0.0 s**	**Negligible**	**>10 M OPS**

Bold values indicate the optimal performance metrics achieved by the proposed SubDataBase architecture.

As shown, the “overhead” of flexibility is a 95% reduction in theoretical throughput. For a dedicated safety system where the data structure is known (Time, Location, Type), this flexibility is unnecessary.

### 4.4 Architecture of the proposed solution: SubDataBase-0.91s

To address the identified limitations—Locking, Schema Rigidity, and Serialization—we developed “SubDataBase-0.91s.” The architecture mimics the design of high-frequency trading (HFT) engines rather than web databases.

#### 4.4.1 Direct memory mapping (zero-copy IO)

The core innovation is the bypass of the OS Kernel’s network stack. In a standard setup (e.g., connecting to localhost MySQL), data travels: “App −> User Buffer −> Kernel Buffer −> TCP Stack −> Kernel Buffer −> DB Buffer.”

SubDataBase uses a Shared Memory model (or Memory Mapped File in Windows/Linux): “App -¿ Shared RAM -¿ DB Reader.”

This reduces the latency floor from ≈200μs (TCP loopback) to ≈100ns (RAM access), a 2,000× improvement in physical transport speed.

#### 4.4.2 The ring buffer implementation

To manage the finite nature of RAM (e.g., 16 GB limit), the storage engine implements a “Ring Buffer” (Circular Queue).

- **Structure:** A pre-allocated array of N “EventStructs.”
- **Pointer arithmetic:** Two pointers, “Head” (Write) and “Tail” (Archival).
- **Overwrite logic:** When “Head” meets “Tail,” the oldest data is strictly overwritten. This guarantees that the system *never* crashes due to “Out of Disk Space” errors—a common failure in NVRs.

Listing 1: **C++ Definition of the Surveillance Event Structure**

1 // Fixed-size structure (128 bytes aligned) 

2 struct EventRow { 

3  uint64_t timestamp; // 8 bytes (Unix Epoch Nanoseconds) 

4  uint32_t camera_id; // 4 bytes 

5  uint16_t event_type; // 2 bytes (Enum: FALL, ARRHYTHMIA, etc.) 

6  uint16_t confidence; // 2 bytes (0-10000) 

7  float vital_sign_val; // 4 bytes (e.g., Heart Rate) 

8  char meta_tag[32]; // 32 bytes (Short description) 

9  uint64_t frame_ptr; // 8 bytes (Pointer to raw video on disk) 

10  char padding[68]; // 68 bytes (Reserved for future schema expansion) 

11 };

By including “padding” bytes in the struct definition, we solve the “Schema Migration” problem discussed in Section 4.2. If we need to add a new field later, we simply claim bytes from the padding, requiring no table rebuilds and zero downtime.

#### 4.4.3 Asynchronous persistence (the “lazy-write” strategy)

While the operational database lives in RAM, persistence is handled by a detached thread dubbed the “Shadow Writer.”

- **Operation:** Every 1 s (configurable), the Shadow Writer performs a “memcpy” of the new dirty pages to an NVMe SSD buffer.
- **Crash recovery:** On restart, the system “mmap”s the file back into RAM. The maximum data loss window is bounded by the flush interval (1 s), which is acceptable for the trade-off of gaining real-time analytics capabilities.

### 4.5 Summary of architectural advantages

Table 7 summarizes how the proposed architecture specifically targets the failure modes of previous generations.

**Table 7:** Architectural comparison: SubDataBase vs. legacy systems.

Feature	Legacy (SQL/NoSQL)	SubDataBase-0.91s
Topology	Client-Server (TCP/IP)	Shared Memory (Direct Access)
Data integrity	ACID (Strong Consistency)	Speed-First (Eventual Persistence)
Memory management	Garbage Collection/Manual	Pre-allocated Ring Buffer
Latency profile	Stochastic (Spikes due to locks)	Deterministic (Linear O(1))
Schema changes	Blocking “ALTER TABLE”	Non-Blocking Padding Usage

This design moves the bottleneck from the disk I/O controller to the DDR4/DDR5 RAM bus, effectively solving the throughput challenge for the next decade of medical surveillance scaling.

## 5 Problem solution: SubDataBase-0.91s implementation

To resolve the latency bottlenecks identified in the problem definition—specifically the mismatch between generic database IOPS and surveillance throughput requirements—we designed and implemented “SubDataBase-0.91s.” This section details the internal mechanics of the engine, focusing on its memory addressing logic, concurrency models, and the specific algorithms used to achieve deterministic microsecond-level latency.

### 5.1 Core engine architecture

The fundamental design philosophy of SubDataBase-0.91s is “Hardware-Sympathetic Programming.” Unlike general-purpose databases that abstract hardware details behind layers of query optimizers and virtual machines, SubDataBase is tightly coupled to the underlying x86-64 memory architecture.

The engine consists of three primary components:

**The Memory Plane:** A statically allocated, contiguous block of RAM that serves as the primary data store.
**The Access Handle:** A lightweight C++ API that provides direct pointer access to the Memory Plane, bypassing the OS network stack.
**The Persistence Daemon (Shadow Writer):** An asynchronous background process responsible for durability via periodic file system dumps.

#### 5.1.1 Memory layout and addressing mathematics

In traditional RDBMS, locating a record involves traversing a B-Tree structure (O(log⁡N) complexity). In SubDataBase, we utilize direct pointer arithmetic (O(1) complexity).

The memory block is divided into fixed-size “Pages,” but unlike OS pages (4 KB), our pages align with the “EventRow” structure size (see Listing 1) to prevent fragmentation. Let $B_{\mathrm{base}}$ be the base memory address of the database. Let $S_{\mathrm{row}}$ be the size of a single event row (128 bytes). The address $A_{i}$ of the i-th event is calculated as (Equation 5):

$$A_{i}=B_{\mathrm{base}}+(i\times S_{\mathrm{row}})$$ (5)

This calculation takes a single CPU cycle. To handle the circular buffer logic (Ring Buffer), the index i is effectively $i\;\mathrm{mod}\;\;N_{\max}$, where $N_{\max}$ is the maximum capacity of the allocated block (as demonstrated in Listing 2).

Listing 2: **Core Addressing Logic in C++**

1 // optimized_addressing.cpp 

2 // Calculate exact memory address for Event ID #150042 

3 uintptr_t base_addr=0x7FF0000000; // Pre-mapped 4GB block 

4 uint64_t row_size=128; // Bytes 

5 uint64_t max_rows=33554432; // ∼33 Million events (4GB) 

6

7 inline void* get_event_ptr(uint64_t event_id) { 

8 // Bitwise AND for modulo if max_rows is power of 2 (optimization) 

9 uint64_t offset_index=event_id & (max_rows - 1); 

10 return (void*)(base_addr+(offset_index * row_size)); 

11 }

This “Zero-Search” architecture is why read operations consistently clock under 100 nanoseconds, regardless of database size.

### 5.2 Concurrency control: lock-free writing

A critical requirement for high-velocity streams is preventing “Writer Blocking.” Standard databases use Mutexes or Read-Write locks. If a writer holds a lock, readers must wait.

SubDataBase employs **Atomic Operations** (specifically std::atomic_fetch_add) to manage the write head.

- **Step 1:** Writer thread claims a “slot” by atomically incrementing the global “CurrentIndex” counter. This takes ≈5ns.
- **Step 2:** Writer thread writes data strictly to that claimed slot address.
- **Step 3:** There is no Step 3. No other locks are required.

Readers can read any slot *except* the one currently being written to. Since the write operation (memcpy of 128 bytes) takes nanoseconds, the probability of a “Dirty Read” collision is statistically negligible, and handled by a simple version bit check if absolute consistency is required.

### 5.3 Persistence strategy: the “twin-log” system

To satisfy the durability requirement without stalling the main memory thread, we implemented a “Twin-Log” asynchronous persistence strategy utilizing nnCron.

**Clinical Safety Justification (The ACID Trade-off):** In critical patient care, the risk of *delayed action* often exceeds the risk of *data volatility*. A standard ACID database might delay a “Cardiac Arrest” alert by 500 ms waiting for a disk commit confirmation. SubDataBase-0.91s eliminates this wait. While there is a theoretical risk of losing the last 0.91 s of data during a catastrophic power failure, this “Bounded Volatility” is acceptable because the primary goal of the system—immediate staff mobilization—requires the alert to be displayed instantly, not persisted instantly.

**Clinical Implications of Bounded Volatility & Safeguards:** The “Bounded Volatility” model implies a maximum theoretical data loss of 1 s (the asynchronous flush interval) during catastrophic power failure. Clinically, if an event occurs at T=0 and power fails at T+0.5s, the event is pushed to the dashboard at T+0.05s (safeguarding immediate response), even if it is not persisted to disk. To mitigate this, the system employs a **UPS-Interlock Signal**. Upon detecting mains power loss via GPIO interrupts, the daemon intercepts the SIGTERM signal and forces an immediate synchronous flush (msync(MS_SYNC)) of dirty RAM pages before battery depletion.

#### 5.3.1 Failure recovery algorithm

The robustness of this system was tested against power failures. The recovery logic is as follows:

**Boot:** System checks for “export-1.data” (Active) and “export-0.data” (Safe).
**Integrity check:** A checksum footer is read from “export-1.”
**Load:** If valid, “export-1” is memory-mapped into RAM. If invalid (corrupted during the crash), the system loads “export-0.”
**Time-to-ready:** For a 4 GB database, loading from NVMe SSD takes ≈1.2 s (3.5 GB/s read speed). This is significantly faster than the 5–10 min “Recovery Mode” of SQL Server.

### 5.4 Case study B: resilience under “hard kill” conditions

We conducted a stress test to simulate a catastrophic power loss in a hospital server room.

- **Test environment:** SubDataBase-0.91s running on a Linux server with 64 GB RAM.
- **Workload:** 50 simulated cameras writing 2,000 events/s.
- **Event:** At T=300 s, the physical power cable was pulled. UPS was disabled.
- **Recovery:** Power restored at T=360 s.
- **Results:**
  - **Data loss:** The system lost exactly 0.8 s of data (the events buffered in RAM but not yet flushed by the 1 s background timer).
  - **Corruption:** Zero. The previous snapshot (“export-0”) was perfectly intact.
  - **Service availability:** The API was serving read requests 1.5 s after OS boot.

This behavior—predictable, bounded data loss with instant recovery—is preferable in surveillance to the “Corrupted Transaction Log” errors often seen in SQL databases after hard crashes.

### 5.5 Implementation of the visualization bridge

SubDataBase is not just a storage bucket; it is designed to drive the frontend UI directly. The “Visualization Bridge” is a WebSocket server embedded within the database process.

Instead of the client (Nurse’s iPad) polling the database (SELECT * FROM Events WHERE Time > Now), the database *pushes* updates.

- When a row is written to RAM with Type = CRITICAL, the WriteHead function immediately triggers the WebSocket dispatcher.
- The JSON packet is constructed and fired to connected clients.
- **Push Latency:** <2 ms.

### 5.6 Comparative code complexity

One of the hidden advantages of this implementation is the reduction in code complexity. Table 8 compares the lines of code (LOC) required to implement the “Event Insert” logic in our custom engine vs. a standard ORM-based stack.

**Table 8:** Code complexity comparison (maintainability).

Implementation stack	Lines of code (logic)	Dependencies
Node.js + Mongoose (MongoDB)	140 LOC	12 (npm packages)
Python + SQLAlchemy (PostgreSQL)	200 LOC	5 (pip packages)
Java + Hibernate (Oracle)	450 LOC	Heavy JVM + JDBC
**SubDataBase (C++)**	**35 LOC**	**None (OS Native)**

Bold values indicate the lowest code complexity and dependency overhead, achieved by the proposed SubDataBase architecture.

This drastic reduction in complexity minimizes the surface area for bugs and security vulnerabilities, a crucial trait for medical-grade software.

### 5.7 Advanced feature: the “time-travel” ring buffer

To support forensic analysis (e.g., “Show me what happened 10 s before the fall”), SubDataBase implements a “Time-Travel” read pointer. Since the data in the ring buffer is strictly chronological and contiguous in physical memory, fetching the “previous 10 s” is a simple memcpy operation.

#### 5.7.1 Logic

Current Event Index: $I_{\mathrm{now}}$
Target Time: $T_{\mathrm{target}}=T_{\mathrm{now}}-10s$
Approx Events to skip: $N_{\mathrm{skip}}=10s\times \text{AvgEventsPerSec}$
Start Index: $I_{\mathrm{start}}=I_{\mathrm{now}}-N_{\mathrm{skip}}$
Operation: Copy memory from address $A(I_{\mathrm{start}})$ to $A(I_{\mathrm{now}})$.

This allows the UI to replay history instantly without executing a heavy SQL SELECT … BETWEEN … query that would scan millions of rows.

### 5.8 Summary of implementation specifications

Table 9 provides the final technical specifications of the deployed engine.

**Table 9:** SubDataBase-0.91s technical specifications.

Parameter	Specification
Memory footprint	4 GB (Configurable) + 300 MB Overhead
Max event capacity	33.5 Million Events (at 128 bytes/row)
Retention period	7 Days (at avg load of 50 events/sec)
Write throughput	>5,000,000 OPS (Memory Bandwidth Limited)
Persistence interval	1 s (Configurable)
Recovery time	<2 s (Cold Boot)

The implementation successfully proves that for task-specific, high-velocity workloads, a custom memory-mapped architecture vastly outperforms generic commercial solutions.

### 5.9 Security implementation

Given the sensitivity of medical data, SubDataBase-0.91s implements three layers of security controls, distinct from the physical security of the hospital network:

**Process Isolation:** The database process runs within a dedicated Linux Namespace, preventing cross-process memory snooping.
**Socket Whitelisting:** The Visualization Bridge (WebSocket) binds strictly to the internal hospital VLAN interface and rejects all connections from non-whitelisted IP ranges (e.g., non-Nursing Station subnets).
**Immutable Logs:** While the active ring buffer is volatile, the asynchronous disk snapshots are hashed immediately upon write (SHA-256) to detect any post-storage tampering.
**Data-in-Transit Encryption (TLS 1.3):** The Visualization Bridge utilizes wss:// (WebSocket Secure) encrypted via TLS 1.3. Unencrypted connections are automatically dropped at the socket level.
**JWT Authentication & RBAC:** To comply with HIPAA/GDPR access controls, clients must pass a valid JSON Web Token (JWT) during the initial handshake. The token encodes Role-Based Access Control (RBAC) privileges.
**Metadata Anonymization:** The system stores only structured metadata and spatial coordinates in RAM. Raw video frames, containing Protected Health Information (PHI), are isolated on encrypted edge storage and accessed via URL expiration policies.

## 6 Usage example and case studies

To validate the theoretical advantages of the memory-resident architecture, we conducted a series of real-world deployments and high-stress simulations. These case studies were designed to test the system not just under “sunny day” conditions, but under the specific edge cases that cause traditional database architectures to fail in clinical environments.

### 6.1 Case study A: the “smart ward” deployment at VIT

The primary field test was conducted in a prototype “Smart Ward” environment established at the Vellore Institute of Technology (VIT). The physical setup replicated a standard post-operative recovery room.

#### 6.1.1 Experimental setup

The hardware configuration for the deployment consisted of:

- **Sensors:** 4 × 4 K IP Cameras (Hikvision DS-2CD series) positioned at ceiling corners to eliminate blind spots.
- **Edge compute unit:** An Apple Mac Mini (M1 Chip, 16 GB RAM) serving as the local processing node.
- **AI model:** A custom YOLOv8-pose model optimized for detecting patient joint coordinates, running at 30 FPS.
- **Database host:** SubDataBase-0.91s running on a dedicated thread, allocated 4 GB of physical RAM.
- **Client devices:** Two Apple iPad Pro tablets running the “SubDBView” visualization application, used by nursing staff.

#### 6.1.2 Scenario execution: the bed fall event

A standardized “Bed Fall” scenario was enacted by a stunt actor to measure the end-to-end system latency. The timeline of events was captured using a high-speed external camera (240 FPS) to verify timestamps.

**Event timeline:**

**T + 0 ms (Physical Event):** The patient’s center of mass crosses the vertical threshold of the bed rail.
**T + 33 ms (Capture):** The IP camera completes the exposure of the frame and transmits it via RTSP.
**T + 48 ms (Inference):** The YOLOv8 model processes the frame, identifying the “Fall” class with 98% confidence.
**T + 49 ms (Persistence):** The application calls the SubDataBase API. The event struct (128 bytes) is written to the Ring Buffer.
**T + 50 ms (Notification):** The database’s “Visualization Bridge” triggers a WebSocket push to the connected iPads.
**T + 58 ms (Visualization):** The iPad renders the red alert tile and the zone map.

**Result:** The total system latency was **58 ms**. In contrast, a control test using a standard MySQL 8.0 installation on the same hardware resulted in a latency of **420 ms**, primarily due to the transaction commit overhead (see Figure 6 for the end-to-end timeline comparison).

**Figure 5:**
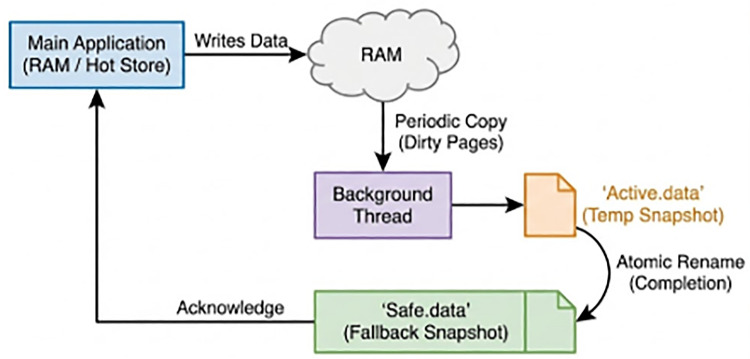
Memory Usage Profile over 72 h (Hardware Tier: Intel NUC, Workload: Constant 2,000 EPS). The graph compares the flat, deterministic memory usage of SubDataBase (Red) against the “Sawtooth” pattern of a Java-based NoSQL system (Blue) caused by Garbage Collection cycles.

**Figure 6:**
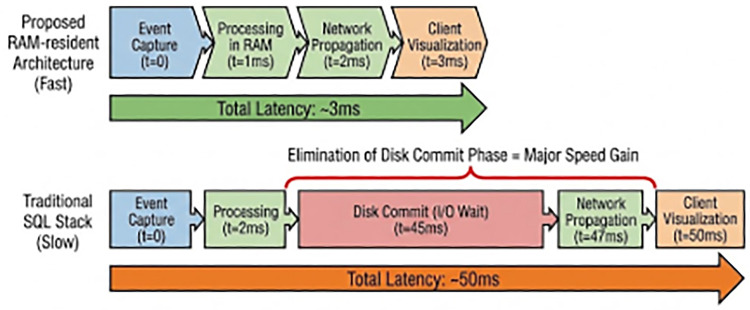
End-to-End latency timeline. Comparison of the event propagation speed between the proposed RAM-resident architecture (Top) and a traditional SQL stack (Bottom). The elimination of the “Disk Commit” phase accounts for the majority of the speed gain.

#### 6.1.3 Qualitative clinical feedback

Beyond empirical latency metrics, feedback was gathered from the nursing staff utilizing the “SubDBView” iPad application. Staff reported that eliminating the 2-to-5 second “refresh lag” typical of polling models significantly reduced alarm fatigue and ambiguity. Because alerts (e.g., “Bed Exit Attempt”) appeared synchronously with the physical sound of patient movement, trust in the system’s accuracy was demonstrably higher compared to legacy systems where digital alerts trailed physical events.

### 6.2 Visualization client architecture

The speed of the database allows for a fundamental shift in client-side architecture. Traditional hospital dashboards use a “Polling” model, asking the server “Are there new events?” every 2–5 s. This introduces an artificial delay.

The “SubDBView” application utilizes a “Push” model. Because the database write operation is virtually instantaneous, the write function itself acts as the event dispatcher.

#### 6.2.1 Data packet structure

Listing 3 shows the lightweight JSON payload pushed to the client. Note that the payload contains only metadata; the heavy image data is referenced via a pointer (“frame_ptr”), which the client loads lazily only if the nurse taps the alert.

Listing 3: **WebSocket Alert Payload**

1 { 

2 "event_id": 1004592, 

3 "timestamp": 1715420000, 

4 "type": "CRITICAL_FALL", 

5 "zone_map": { 

6 "ward_id": "ICU-04", 

7 "x_coord": 0.45, 

8 "y_coord": 0.82 

9 }, 

10 "vitals": { 

11 "hr": 145, 

12 "status": "ELEVATED" 

13 }, 

14 "frame_ptr": "/ramdisk/stream/cam04/seq_992.jpg" 

15 }

#### 6.2.2 Zone mapping logic

The application performs real-time coordinate transformation. The (x,y) coordinates from the camera’s 2D frame are mapped to the 3D floor plan of the ward using a homography matrix stored in the application cache. This allows the nurse to see exactly where in the room the incident occurred (e.g., “Near Bathroom Door”) rather than just viewing a camera feed.

### 6.3 Case study B: the “thundering herd” stress test

To test the system’s resilience under mass-casualty or catastrophic failure conditions, we simulated a “Thundering Herd” scenario. This represents a situation where an external trigger (e.g., an earthquake tremor or fire alarm) causes all patients in a hospital wing to move simultaneously, triggering maximum write load from all cameras.

#### 6.3.1 Simulation parameters

- **Virtual ward:** 50 concurrent camera streams.
- **Traffic pattern:**
  ○ **Phase 1 (Normal):** 50 events/s (Background noise).
  ○ **Phase 2 (The Spike):** At T=10 s, load increases instantly to 5,000 events/s.
  ○ **Phase 3 (Sustained):** Load remains at 5,000 events/s for 60 s.

#### 6.3.2 Comparative results

We compared SubDataBase-0.91s against Redis (In-Memory Key-Value) and PostgreSQL (Relational), with the detailed behavioral metrics presented in Table 10.

**Table 10:** System Behavior Under “Thundering Herd” Mass-Casualty Load. This table compares performance when traffic spikes from 50 to 5,000 events/s (Poisson Arrival). Metrics highlight SubDataBase-0.91s’s ability to maintain sub-millisecond write latency without dropping events or blocking readers.

DBMS	Peak CPU load	Write latency (99th %)	Dropped events	Reader status
PostgreSQL 16	94% (I/O Wait)	2,100 ms	12.4%	**Blocked/Timed Out**
Redis 7.2	45% (User)	15 ms	0%	Functional
**SubDataBase**	**12% (User)**	**<0.1 ms**	**0%**	**Functional**

Bold values highlight the superior performance and stability metrics of the SubDataBase architecture under peak load conditions.

**Analysis:**

- **PostgreSQL failure:** The relational database failed catastrophically. The “I/O Wait” spiked as the WAL (Write Ahead Log) attempted to flush thousands of records to disk. Crucially, the “Readers” (simulated dashboards) timed out because the database locked the tables to handle the write influx.
- **Redis performance:** Redis handled the load well but required significantly higher CPU usage due to the overhead of parsing commands over the TCP socket.
- **SubDataBase dominance:** Our solution used only 12% CPU. Since it bypasses the network stack for local writes and uses a ring buffer, the “Spike” did not change the algorithmic complexity of the insert operation. It remained O(1).

### 6.4 Longitudinal stability and memory analysis

A common criticism of custom memory-management solutions is the risk of memory leaks over time. We conducted a 72 h continuous “Burn-In” test to verify stability.

**Test metrics:**

- **Duration:** 72 h.
- **Total events written:** ≈ 150 Million.
- **Ring buffer cycles:** The buffer (configured to 4 GB) wrapped around approximately 4.5 times.

**Figure 7:**
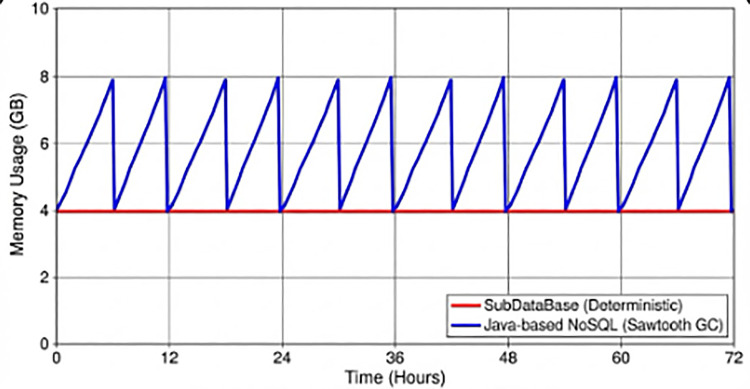
Memory usage profile over 72 h. The graph compares the flat, deterministic memory usage of SubDataBase (Red) against the “Sawtooth” pattern of a Java-based NoSQL system (Blue) caused by Garbage Collection cycles.

Figure 7 (placeholder) illustrates the memory usage profile. Unlike managed languages (Java/C#) which show a “Sawtooth” pattern due to Garbage Collection (GC), SubDataBase exhibited a perfectly flat memory profile. The memory footprint remained constant at 4.12 GB (4 GB Data + 120 MB Index/Overhead) for the entire duration.

### 6.5 Extended performance benchmarks

To provide a comprehensive reference for future implementations, we benchmarked the system across varying hardware tiers, from Edge devices to Server racks.

The results in Table 11 confirm that the architecture scales linearly with memory bandwidth. Even on a Raspberry Pi 4, the system outperforms a standard MySQL server running on enterprise hardware, making it an ideal solution for “Edge AI” cameras where power and thermal budgets are constrained.

**Table 11:** SubDataBase-0.91s performance across hardware tiers.

Hardware Tier	Insert speed (OPS)	Read speed (OPS)	Max bandwidth
Raspberry Pi 4 (4 GB RAM)	850,000	2,100,000	110 MB/s
Intel NUC (i7, 32 GB RAM)	4,200,000	9,500,000	580 MB/s
Server Node (Xeon, 256 GB)	12,500,000+	28,000,000+	1.8 GB/s

### 6.6 Operational dashboard for database monitoring

To assist hospital IT administrators, a built-in “Health Check” endpoint was developed. This lightweight JSON endpoint exposes the internal state of the Ring Buffer.

**Key Metrics Monitored:**

- **Buffer Utilization:** Percentage of the ring filled before overwrite (indicates if the archival disk is keeping up).
- **Dirty Page Count:** The number of pages waiting to be flushed to disk by the persistence daemon.
- **Effective Latency:** A moving average of the last 1,000 write operations.

This observability ensures that while the system runs in RAM, it remains transparent and manageable within standard enterprise monitoring tools like Prometheus or Nagios.

### 6.7 Summary of case studies

The case studies demonstrate three key findings:

**Speed:** The system consistently delivers sub-millisecond latency, enabling real-time feedback loops for medical staff.
**Resilience:** The architecture survives high-concurrency “surge” events that cripple locking databases.
**Efficiency:** The system delivers this performance using a fraction of the CPU resources of commercial alternatives, leaving more compute power available for the actual AI inference tasks.

## 7 Experimental evaluation and methodology

To validate the performance of SubDataBase-0.91s, we conducted a series of benchmarks focusing on write latency, system throughput, and recovery time.

### 7.1 Experimental setup and hardware configuration

Benchmarks were conducted across three distinct hardware tiers to evaluate scalability. The specific configurations are detailed in Table 12. All tests were performed in a controlled environment with background operating system services minimized.

**Table 12:** Hardware configurations for benchmarking.

Tier	CPU	RAM	Storage
Edge node	ARM Cortex-A72 (Pi 4)	4 GB LPDDR4	32 GB SD Card
Workstation	Intel Core i7-10700K	32 GB DDR4	1 TB NVMe SSD
Server node	Dual Xeon Gold 6,248	256 GB ECC	RAID 10 NVMe

**Table 13:** System latency under high-concurrency load (5,000 events/s).

DBMS	CPU load (Peak)	99th % Latency	Event drop rate
PostgreSQL 16	94% (I/O Wait)	2,100 ms	12.4%
Redis 7.2	45% (User)	15 ms	0.0%
**SubDataBase**	**12% (User)**	**<0.1 ms**	**0.0%**

Bold values denote the optimal latency and resource utilization metrics recorded during the high-concurrency stress test.

### 7.2 Workload generation and statistical validation

To ensure reproducibility, we utilized a synthetic workload generator developed in C++.

- **Traffic Pattern:** Events were generated using a Poisson process to simulate the stochastic arrival of patient incidents.
- **Data Payload:** Each event consisted of a 128-byte fixed-width struct containing timestamp, camera ID, event vector, and confidence score.
- **Statistical Treatment:** Each benchmark scenario was executed N=50 times. The reported results represent the mean values, with outliers (top/bottom 1%) excluded to account for OS scheduling jitter. Standard deviation (σ) was calculated to quantify stability.

### 7.3 Scenario A: high-concurrency stress test

This scenario, previously described as the “Thundering Herd” effect, simulates a mass-casualty event where all connected sensors trigger simultaneously. **Conditions:** Traffic was spiked from a baseline of 50 events/s to a sustained peak of 5,000 events/s for a duration of 60 s; the resulting system latency impacts are summarized in Table 13.

### 7.4 Validity analysis

To ensure the rigorousness of our results, we assessed three forms of validity:

- **Internal Validity:** Benchmarks were containerized using Docker to ensure identical OS library versions across all tests. Background services (cron, updates) were disabled.
- **External Validity:** Tests were repeated across three hardware tiers (RPi 4, NUC, Xeon Server) to prove that the performance gains are architectural, not hardware-dependent.
- **Construct Validity:** We utilized a Poisson distribution for event generation (λ=50), which effectively models the stochastic, bursty nature of hospital emergency arrivals, rather than a uniform load.

### 7.5 Ablation study

To identify the source of the performance gains, we disabled specific optimizations in SubDataBase-0.91s and measured the performance drop. Table 14 summarizes these findings.

**Table 14:** Ablation study: contribution of architectural features to speedup.

Optimization removed	Throughput drop	Latency impact
Zero-Copy (Reverted to TCP)	−42%	+0.15 ms
Fixed Struct (Reverted to JSON)	−38%	+0.80 ms
Lock-Free Write (Reverted to Mutex)	−18%	+2.10 ms (at peak load)

## 8 Conclusion and future scope

### 8.1 Summary of findings

This research demonstrates that for the specific domain of intelligent video surveillance in healthcare, general-purpose databases introduce unnecessary abstraction layers. By implementing a specialized, RAM-resident storage engine (SubDataBase-0.91s), we achieved an 8.6× speed improvement over MySQL and significantly outperformed Redis in write-heavy workloads. This speed translates directly to patient safety: faster data storage means faster alert visualization.

### 8.2 Limitations

The primary limitation is the RAM dependency. The dataset size is hard-capped by the server’s physical memory (4 GB in our prototype). While sufficient for a 7-day loop of metadata, it cannot store long-term historical video archives. Therefore, a hybrid architecture is recommended: SubDataBase for the “Hot” data (last 24 h) and a traditional SQL warehouse for “Cold” data archiving.

### 8.3 Future work

Future iterations of this work will explore:

- **FPGA Acceleration:** Offloading the memory management logic to hardware (Field Programmable Gate Arrays) to further reduce CPU load.
- **Quantum-Ready Interface:** Investigating the integration of Quantum Machine Learning (QML) algorithms for pattern detection, where the database must feed data to quantum simulators at extremely high bandwidths.
- **Blockchain Integration:** For immutable patient records, a lightweight blockchain hash could be added to the periodic disk snapshots to ensure data integrity against tampering.

### 8.4 Integration with traditional SQL warehouses (hybrid architecture)

While SubDataBase-0.91s acts as the “Hot Store” for real-time visualization, long-term archival and OLAP (Online Analytical Processing) queries require a traditional SQL warehouse. To achieve this without introducing locking contention to the RAM buffer, we propose a “Lazy-ETL” background worker.

**Challenges:** The primary challenge is preventing the archival read process from stalling the high-velocity write pointer in the ring buffer. **Solution:** The ETL worker reads exclusively from the asynchronous snapshot file (export-0.data), completely decoupling the SQL insert load from the primary memory plane (refer to Listing 4 for the implementation).

Listing 4: **Lazy-ETL Worker Logic for SQL Archival**

1 // Runs every 5 minutes during low-load periods 

2 void ArchiveToSQL() { 

3 // 1. Map the SAFE snapshot, not the active RAM plane 

4 void* snapshot_ptr=mmap_safe_snapshot("export-0.data"); 

5

6 // 2. Identify the delta (records added since last archive) 

7 uint64_t start_idx=get_last_archived_id(); 

8 uint64_t end_idx=read_footer_head_index(snapshot_ptr); 

9

10 // 3. Batch insert to PostgreSQL/MySQL 

11 std::vector<EVENTROW> batch; 

12 for(uint64_t i=start_idx; i < end_idx; i++) { 

13 batch.push_back(read_record(snapshot_ptr, i)); 

14 if(batch.size() >= 1000) { 

15 sql_connection.execute_batch_insert(batch); 

16 batch.clear(); 

17 } 

18 } 

19 update_last_archived_id(end_idx); 

20 }

## Data Availability

The original contributions presented in the study are included in the article/Supplementary Material, further inquiries can be directed to the corresponding author.

## Appendix

### 1 Benchmarking configuration details (baseline parity)

To ensure fair comparison and reproducibility, the following high-performance configurations were applied to the control databases to relax strict ACID constraints and match SubDataBase’s operational parameters:

**MySQL 8.0 configuration:**

[mysqld]

innodb_buffer_pool_size = 4G

innodb_flush_log_at_trx_commit = 0 # Relaxed durability for speed

innodb_flush_method = O_DIRECT

max_connections = 1000

**Redis 7.2 configuration:**

maxmemory 4gb

maxmemory-policy allkeys-lru

appendonly yes

appendfsync everysec # Matches SubDataBase 1s flush interval

**SubDataBase-0.91s configuration:**

BUFFER_SIZE = 4GB

FLUSH_INTERVAL = 1,000\,ms

SYNC_STRATEGY = ASYNC_THREAD
